# A Longitudinally Extensive Spinal Cord Lesion Restricted to Gray Matter in an Adolescent Male

**DOI:** 10.3389/fneur.2019.00270

**Published:** 2019-03-20

**Authors:** Danielle Golub, Faith Williams, Taylor Wong, Nishanth Iyengar, Hannah Jolley, Sakinah Sabadiah, David Rhee, Gabrielle Gold-von Simson

**Affiliations:** ^1^New York University School of Medicine, New York, NY, United States; ^2^School of Medicine, Washington University School of Medicine, Saint Louis, MO, United States; ^3^Department of Pediatrics, New York University School of Medicine, New York, NY, United States; ^4^Department of Neurology, New York University School of Medicine, New York, NY, United States; ^5^Health and Hospitals, Clinical Translational Science Institute, New York University, New York, NY, United States

**Keywords:** myelitis, gray matter, anterior horn, ADEM, acute flaccid myelitis

## Abstract

Longitudinally extensive spinal cord lesions (LECL) restricted to gray matter are poorly understood as are their neurodevelopmental repercussions in children. We herein report the critical case of a 13-year-old male presenting with progressive quadriparesis found to have cervical LECL restricted to the anterior horns. Challenged with a rare diagnostic dilemma, the clinical team systematically worked through potential vascular, genetic, infectious, rheumatologic, and paraneoplastic diagnoses before assigning a working diagnosis of acute inflammatory myelopathy. Nuanced consideration of and workup for both potential ischemic causes (arterial dissection, fibrocartilaginous embolism, vascular malformation) and specific inflammatory conditions including Transverse Myelitis, Neuromyelitis Optica Spectrum Disorders (NMOSD), Multiple Sclerosis (MS), Acute Disseminated Encephalomyelitis (ADEM), and Acute Flaccid Myelitis (AFM) is explained in the context of a comprehensive systematic review of the literature on previous reports of gray matter-restricted longitudinally extensive cord lesions in children. Treatment strategy was ultimately based on additional literature review of treatment-refractory acute inflammatory neurological syndromes in children. A combination of high-dose steroids and plasmapheresis was employed with significant improvement in functional outcome, suggesting a potential benefit of combination immune-modulatory treatment in these patients. This case furthermore highlights quality clinical reasoning with respect to the elusive nature of diagnosis, nuances in neuroimaging, and multifocal treatment strategies in pediatric LECL.

## Introduction

Longitudinally extensive spinal cord lesions (LECL) are a phenomenon of rapidly progressive and wide-spread spinal cord inflammation with potentially devastating clinical consequences. While LECL is best described in the literature as a key diagnostic criterion in Neuromyelitis Optica Spectrum Disorders (NMOSD), it is also an infrequent, but critical, sequela of a broad range of inflammatory central nervous system syndromes (1, 2). Inflammatory LECL is most commonly described as an autoimmune-mediated swelling of myelin sheaths directed by anti-myelin basic protein (MBP) antibodies, anti-aquaporin 4 (AQP-4) antibodies, and anti-myelin oligodendrocyte glycoprotein (MOG) antibodies, among others (3). Gray matter involvement in LECL, although rare in early manifestations of disease, is an acute finding indicative of either more progressive and refractory presentations of common autoimmune syndromes (multiple sclerosis, acute disseminated encephalomyelitis, NMOSD, sarcoidosis), or under-recognized entities such as pediatric peri-infectious acute flaccid myelitis (AFM) and paraneoplastic syndromes (4–9).

LECLs demonstrate abnormal hyperintense signal on T2-weighted magnetic resonance imaging (MRI) in the spinal cord that spans at least three vertebral segments (1). Differentiating between lesions to make a diagnosis can be complicated by the ambiguity of MRI findings among inflammatory, infectious, and vascular etiologies. In particular, gray matter-restricted LECL may closely resemble anterior spinal artery (ASA) infarction, simulating the “owl's eye” or “snake eye” appearance on T2-weighted axial MRI (10). A prospective study comparing imaging in early neuromyelitis optica spectrum disorders (NMOSD) and ASA infarction found that the “owl's eye” sign tended to complicate the diagnosis (6). Furthermore, a variable and indeterminate appearance of centrally located, T2-hyperintense LECL has been described in both MOG and AQP-4-related NMOSD, infarction, viral myelitis, sarcoidosis, and spondylitic compressive myelopathies, and most recently in paraneoplastic disorders such as GFAP autoimmunity (11–14). In pediatric patients especially, LECL limited to the gray matter generates a non-specific differential diagnosis and imparts a risk of delayed diagnosis and possible long-term morbidity (3, 15, 16). The following case of a pediatric patient with an acute and debilitating presentation of LECL sharply restricted to the gray matter illustrates the importance of a multidisciplinary, clinical reasoning team approach to diagnosis and treatment in an elusive and rare pediatric lesion.

## Case Report

### History

A 13-year-old male presented to the Pediatric Emergency Room at a tertiary care academic medical center due to progressive quadriparesis that reportedly began after a prolonged episode of coughing 3 days earlier. His neurological symptoms manifested as bilateral hand numbness with persistent back and neck pain, but rapidly progressed to quadriparesis and widespread tactile and proprioceptive sensory loss over 2 days. The day prior to his presentation, he was unable to walk without support. He was subsequently admitted to the Pediatric Intensive Care Unit (PICU) due to concern for potential rapid respiratory compromise.

Other than a cough, the patient reported no recent acute illness and denied travel, trauma, exposures, and vaccinations (all childhood vaccinations were up to date). Additionally, there was no history of developmental or cognitive impairment (was performing well in eighth grade), no drug or alcohol use, and no history of smoking or e-cigarette use. He had a history of mild persistent asthma, and his only medication was his albuterol inhaler that he had been using about twice daily for a week prior to admission. There were no reported allergies.

### Presentation

At the time of admission, he was hemodynamically stable with normal vital signs. He was afebrile and in no acute distress. Heart and lung exams were normal. Neurological exam revealed diffuse hypotonia, diminished deep tendon reflexes in all extremities, persistent quadriparesis with most marked weakness in the radial, median, and ulnar nerve distributions (C5-T1 levels), and decreased sensation at the C4-L2 levels. Additionally, the patient had diffuse, severe hyperesthesia in response to sharp stimuli. There was no evidence of acutely altered mental status, visual or other cranial nerve deficit, nystagmus, or overt ataxia. Babinski and Hoffman's signs were negative and there was no ankle clonus. There was no spinal or paraspinal tenderness to palpation.

### Hospital Course

On hospital day (HD) 1, still unable to support himself while standing, the patient developed urinary retention requiring multiple straight catheterizations, representing a symptomatic nadir 2–3 days after initial symptom onset. He otherwise remained hemodynamically and neurologically stable during his stay in the PICU. He showed steady recovery after the following workup and treatment on HD 3–10 and was discharged to the Pediatric Acute Rehabilitation Unit on HD 22 in good condition.

#### Initial Diagnostic Studies

**Figure 1:**
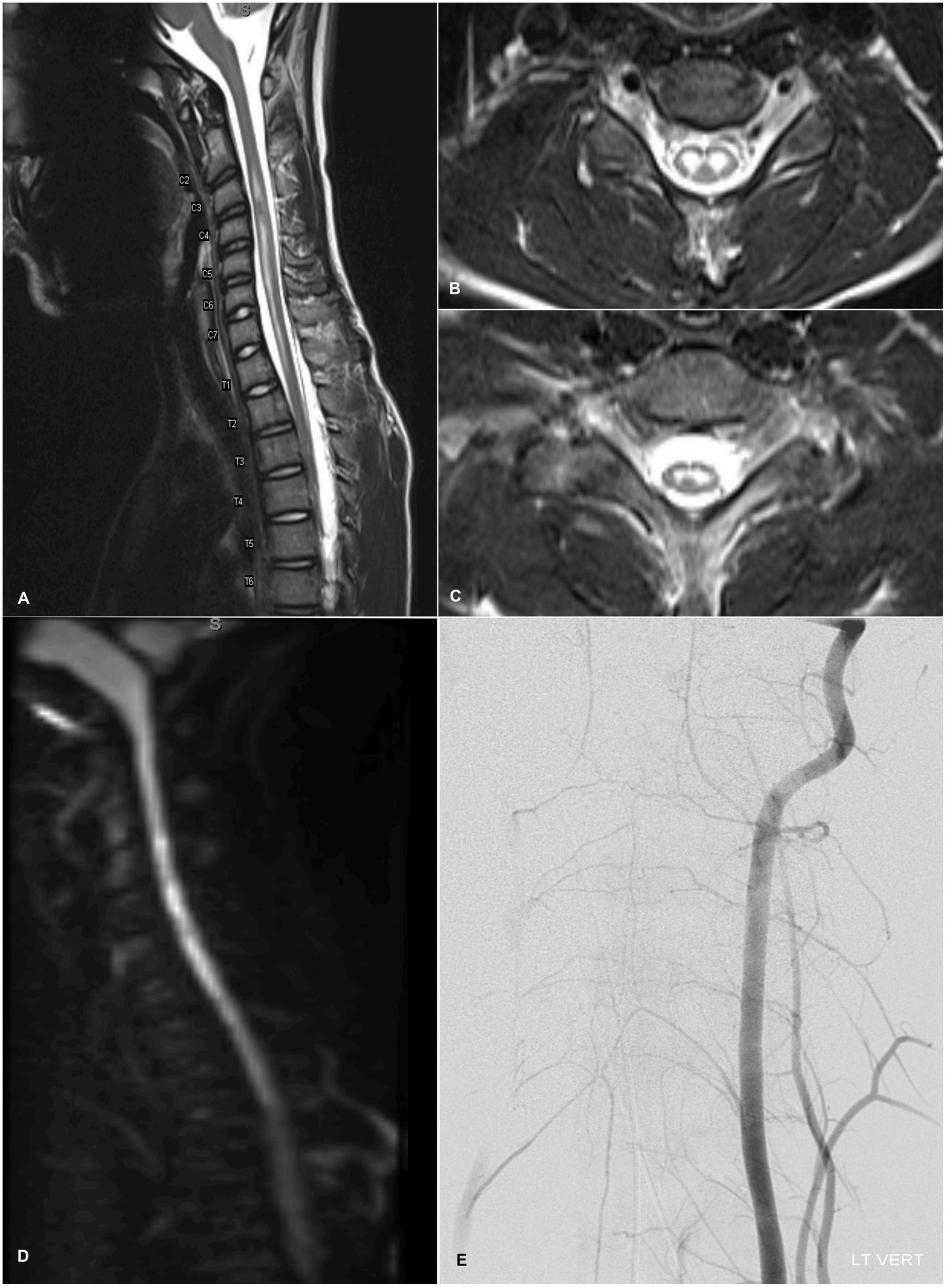
**(A)** T2-weighted sagittal admission MRI image of the cervical spine showing longitudinally extensive, non-enhancing, mildly expansile hyperintensity within the central gray matter of the spinal cord spanning levels C2-T2 with normal appearance of the vertebral flow voids. Mild intervertebral disc intensity changes are observable at C2-C6. **(B,C)** Axial T2-weighted MRI images at levels C6 and C8 respectively demonstrating localization of T2-hyperintensity to the anterior horns with an “owl's eye” appearance. **(D)** Diffusion-weighted imaging demonstrating diffusion restriction within the cervical cord lining up with the T2-hyperintense lesion. **(E)** Diagnostic angiogram showing the cervical view of the left vertebral artery catheterization. Brisk and robust collateralization to the vascular supply of the spinal cord, including the anterior spinal artery, and spinal canal is seen. There is no evidence of infarction or dissection of the spinal vasculature.

Given the acute nature of the patient's neurological symptoms, on HD 1 brain and spine imaging were performed. Brain CT and MRI were unremarkable—there were no lesions, structural abnormalities, or edematous changes of the parenchyma or cranial nerves, and there was no contrast enhancement or leptomeningeal disease. Total spine MRI, however, showed a longitudinally extensive, non-enhancing, T2-hyperintense central cord lesion strictly involving the gray matter from C2-T2 with mild edema, intervertebral disc intensity changes in the cervical levels (Figures 1A–C) and robust diffusion restriction (Figure 1D). These findings were initially thought to be suggestive of spinal cord infarct, although no abnormal flow void, cord compression, mass lesion, discrete disc herniation or desiccation, or other spinal or foraminal stenosis was noted. Follow-up MRA of the thoracic aorta was inconclusive, and concern for spinal ischemia necessitated high-resolution intravascular imaging. On HD 3, interventional angiogram showed brisk, robust filling and collateralization of the vascular supply of the spinal cord, and canal after left vertebral artery injection (Figure 1E). The anterior spinal artery was patent and of normal caliber. There was no evidence for aortic dissection or other vascular abnormality. Furthermore, serial fibrinogen, coagulation, and liver function tests showed no evidence of coagulopathy.

#### Further Work Up and Treatment

Out of secondary concern for an autoimmune or infectious etiology, the patient was started on Intravenous Immunoglobulin (IVIG) (400 mg/kg daily), high-dose steroids (methylprednisolone 1 g daily), vancomycin, ceftriaxone, and acyclovir for empiric meningoencephalitis coverage, and aspirin (81 mg daily) for secondary prevention of possible cord ischemia. Initial laboratory workup, however, was not indicative of an infectious or an acute inflammatory etiology with a normal white blood cell count (7.1^*^10^3^/μL), negative blood cultures and respiratory viral panel, and normal sedimentation rate and C-reactive protein levels (1 mm/h and <0.1mg/L, respectively). Urine studies, including toxicology, were likewise unremarkable. Cerebrospinal fluid (CSF) obtained by lumbar puncture also proved unrevealing (WBC 0, RBC 9, glucose 65, protein 27.3). CSF infectious studies for enterovirus, HSV 1/2, syphilis, Lyme disease, ehrlichiosis, Cryptococcus, Epstein-Barr virus, and cytomegalovirus were negative, and serum titers for additional pathogens including mycoplasma and West Nile virus did not support acute infection. Additional workup for common autoimmune myelopathies including neuromyelitis optica spectrum disorders (NMOSD) and multiple sclerosis (MS) was non-diagnostic (serum was negative for aquaporin-4 antibodies (AQP-4) (titer <1:10), myelin oligodendrocyte glycoprotein antibodies (MOG) (titer <1:10), and CSF was negative for oligoclonal bands). Serum tests for myositis (aldolase, creatine kinase) were negative.

#### Recovery and Follow Up

After two doses of high-dose steroids (total 5 day course) and IVIG on HD 3–5, the patient's lower extremity strength began to improve, urinary retention resolved, and deep tendon reflexes returned to baseline. Given the negative infectious workup, antibiotics and acyclovir were discontinued on HD 3. However, the patient demonstrated a persistent weakness of the bilateral upper extremities that prompted a 5 day plasmapheresis course (HD 5–10) as salvage therapy for medically-refractory myelitis. Over the next few days, the patient achieved significant gains in strength, sensation, and balance.

The patient was transferred to a pediatric acute rehabilitation unit on HD 22. His exam remained notable for diminished hand, wrist, biceps, and triceps strength bilaterally, but he was able to walk with minimal assistance and his muscle strength normalized. Sensation was intact in the extremities but remained slightly diminished in the T4-T10 dermatomes. Paresthesias were no longer present. At his 3 month follow-up with the pediatric neurologist, his only residual finding was bilateral hand weakness, although greatly improved. He was back at school on a full-day, regular schedule. Diagnosis remained “unspecified inflammatory spinal cord syndrome.”

## Discussion

LECL is a rare phenomenon and presents a critical diagnostic dilemma in pediatric patients. Possible etiologies for LECL include traumatic, ischemic, vascular, inflammatory, and infectious causes or even a genetic polymorphism, each requiring rapid diagnosis and unique treatment for successful recovery (17). LECLs are well-described as a characteristic features of NMOSD. However, in contrast to this patient, the lesions of NMOSD are typically centrally located, diffuse or patchy, extend to the medulla, demonstrate contrast enhancement, and are only rarely seen restricted solely to the anterior horns (18, 19). Furthermore, most cases of NMOSD and transverse myelitis are associated with a central, expansive, and contrast-enhancing lesion (20, 21). Our patient's presentation was particularly enigmatic in that his LECL was isolated to the anterior horns yielding the “owl's eye” appearance on T2-weighted imaging with restricted diffusion but without gadolinium enhancement. Initial unfamiliarity with the potential diagnoses in a non-traumatic pediatric acute anterior spinal cord myelopathy resulted in delayed diagnosis and the use of numerous empiric treatments. A systematic review of PubMed/MEDLINE and EMBASE for reports sharing MRI findings and pathology consistent with gray matter-restricted LECL in pediatric patients is shown in Table 1. The search strategy used in PubMed is defined in Appendix A and was adapted for EMBASE.

**Table 1:** Previous Reports of Isolated Longitudinally Extensive Gray Matter Cord Lesions in Pediatrics.

	**Age/Gender**	**Symptoms**	**MRI Findings**	**Diagnosis**	**Treatment(s)**
Ghosh and Mitra (22)	10yo M	Post-traumatic acute onset neck pain, quadriparesis, bladder, and bowel dysfunction	T2-hyperintensity of anterior horns from levels C3-C8	Fibrocartilaginous Embolism/Spinal Cord Ischemia	Steroids
Monden et al. (23)	14yo M	Post-viral acute onset of weakness in of all four extremities, urinary retention, bowel dysfunction, T10 sensory level, decreased proprioception	T2-hyperintensity of central gray matter from C2-T11 and satellite lesions in the ventral medulla and pons	ADEM	Acyclovir, IVIG, Methylprednisolone, Mannitol boluses
Reisner et al. (24)	8yo F, 8mo F, 12yo F	Acute onset neck pain, paresthesias, weakness in various extremities, decreased tactile sensation, onset of symptoms post-fall	Cervical to thoracic spinal cord swelling on T2-weighted images localized to gray matter with diminished signal in multiple disc spaces. Restricted diffusion of T2-hyperintense areas	Fibrocartilaginous Embolism / Spinal Cord Ischemia	Methylprednisolone, Plasmapheresis
DeSena et al. (25)	14yo M	Rapidly progressive flaccid paralysis and burning pain of the bilateral lower extremities with severe urinary retention	Increased intramedullary T2 signal localized to anterior horns from levels T11 to the conus medullaris. Diffuse enhancement of ventral nerve roots	Idiopathic Transverse Myelitis	IVIG, High-Dose Steroids, Cyclophosphamide, Plasmapheresis
Amaral et al. (26)	9yo F	Headache, somnolence, meningismus, flaccid paraparesis, pain, and vibratory hypoesthesia at T2, and cervical adenopathy	Hyperintensity in the central cord on T2-weighted imaging localized to the anterior and lateral horns spanning T2-T10	EBV-related Transverse Myelitis	IVIG, Ganciclovir, Valganciclovir
Elpers et al. (27)	12yo F	Acute onset paresis of left upper extremity and progressive paresis of left lower extremity with meningismus and dizziness	Longitudinally-extensive T2-weighted hyperintensity localized to gray matter from levels C2-T2	AQP4-Positive NMOSD	Methylprednisolone, Prednisone taper, Cefotaxime, Acyclovir, Plasmapheresis, Azathioprine, Rituximab
Nelson et al. (28)	17yo F	Left hip pain and weakness, left lower extremity paresthesias with decreased sensation throughout, eventually developed flaccid paralysis	Increased T2 signal in anterior cord at T11-L1 with notable disk extrusion at T10-T11. Focal restricted diffusion in anterior horns noted	Spinal Cord Ischemia	Dexamethasone, Aspirin, Methylprednisolone, Vitamin B12
Esposito et al. (29)	4yo M	Post-viral acute onset of generalized weakness and meningismus, hypotonia and areflexia of left arm	T2-hyperintensity of anterior horns of cervical spinal cord. Slight enhancement of caudal roots without cord enhancement	AFM (enterovirus-D68)	Methylprednisolone, Plasmapheresis, IVIG, Prednisone taper
Girard et al. (30)	4yo F	Acute onset of fever, tetraparesis and urinary retention	T2-hyperintensity of anterior horns extending from cervicomedullary junction to T10 without gadolinium enhancement	Biotinidase Deficiency (first misdiagnosed as NMOSD)	Methylprednisolone, Plasmapheresis, Rituximab, Biotin
Hayashi et al. (31)	5yo F	Acute asthma exacerbation followed by sudden onset bilateral lower extremity paralysis	T2-hyperintensity of anterior and posterior horns and edema at T11-L1	Hopkins Syndrome (enterovirus-D68)	IVIG, Methylprednisolone, Ampicillin/Sulbactam
Hsu et al. (32)	12yo M	Acute onset lower back pain, paralysis and numbness of all four extremities, altered mental status, hyperalgesia, and bladder dysfunction	Longitudinally-extensive gray matter hyperintensity and swelling from C3-T1 and in the conus medullaris	MGUS-associated Transverse Myelitis	Methylprednisolone, Plasmapheresis
Hu et al. (33)	19yo F	Hypotonia of lower extremities, loss of lower extremity reflexes, whole-body paresthesias, meningismus	Diffuse swelling and high intensity signal in medulla, thoracic cord. Owl's eye sign (bilateral anterior horn hyperintensities) on T2-weighted image of thoracic cord	SLE	IVIG, Methylprednisolone, Cyclophosphamide, Aspirin
Yoder et al. (34)	8yo M	Cough, meningismus, altered mental status, anorexia, abdominal pain, right arm paresthesias, and areflexia	T2-hyperintensity of central cervical cord extending up to the cervicomedullary junction with subtle involvement of the pons, midbrain, and cerebellum	AFM (enterovirus-D68)	Ceftriaxone, Acyclovir, Methylprednisolone, IVIG
Chen et al. (35)	5yo M	Hypotonia and areflexia of left upper extremity, winged left scapula, meningismus	T2-hyperintensity of anterior horns from C1-T5	AFM (enterovirus-D68)	IVIG, Methylprednisolone, Ciprofloxacin, Ceftriaxone, Prednisone taper
Wang et al. (36)	13yo M, 13yo M	Subacute onset of bilateral lower extremity weakness and bowel/bladder incontinence, meningismus, foot drop, hyperreflexia	LETM with abnormal T2 signal most pronounced in anterior gray matter with “owl's eye” appearance	Anti-MOG antibody syndrome	Methylprednisolone, Plasmapheresis, IVIG

Our patient's history of a coughing paroxysm, back pain, and clinician awareness of the more common association of the “owl's eye” radiographic sign and restricted diffusion with ischemia (reflected by the greatest number of cases in Table 1) prioritized aortic dissection or other vascular pathology on the differential. Dissection and vascular malformation were ruled out by angiogram, but transient ischemic injury from a clot or fibrocartilaginous embolism dislodged after the prodromal coughing paroxysm remained under consideration. One of the largest case series of patients with suspected fibrocartilaginous embolism reported normal angiography in nearly all patients for whom imaging was available (37). While no explicit evidence of disc desiccation or herniation was seen, intervertebral disc intensity changes in our patient's cervical spine on T2-weighted imaging may be suggestive of premature disc disease, a critical risk factor for fibrocartilaginous embolism (38, 39). Furthermore, the risk of fibrocartilaginous embolism is thought to be elevated in children due to increased presence of small spinal arteries that would allow for retrograde migration of emboli (38, 40). Our patient's presentation is also generally congruent with recently proposed diagnostic criteria for spinal cord infarction: while symptoms worsened in a stepwise manner over 2–3 days and not within the expected 12 h timeframe, CSF was non-inflammatory and spinal MRI showed diffusion restriction without cord compression (41). Although fibrinogen and other coagulation testing was negative, given the heightened concern for possible embolic ischemic injury, the patient remained on aspirin throughout his hospitalization.

Infectious or inflammatory etiologies were also highly considered, especially since the patient reported recent cough. Since 2014, the incidence in the United States of a virally-induced spinal cord disease known as AFM has slowly increased (8). Much like our patient, AFM presents with acute flaccid limb weakness following a viral prodrome and MRI findings of LECL on T2-weighted imaging largely restricted to the anterior horns with a predilection for the cervical spine (8, 42) Positive serum, CSF or respiratory viral panel for enteroviruses D68 and 71 are heavily associated with recent cases, but AFM historically has been linked to a host of viruses including West Nile, coxsackie, and adenovirus (8) Furthermore, there are a handful of reports of a heterogenous entity linking asthma exacerbation and AFM in children, Hopkins Syndrome, most of which describe similar imaging findings as in our patient (31, 43, 44). However, in our patient, a causative viral agent or definitive prodrome was not identified and therefore a diagnosis of AFM could not be made.

Further workup for a possible inflammatory etiology included testing for AQP-4 and MOG-associated NMOSD, multiple sclerosis (MS), and other autoimmune disorders. MOG antibody-associated myelitis is associated with gray-matter restricted T2-signal changes coupled with a lack of gadolinium enhancement and an AFM-like phenotype, as was seen in our patient (45). Serum anti-MOG and anti-AQP4 tests, however, were negative and are known to have a high degree of sensitivity in NMOSD and other anti-MOG syndromes (46, 47). It is important to note, however, that our patient received two treatments of intravenous immunoglobulin (IVIG) prior to AQP-4 and MOG antibody testing, which is known to diminish test sensitivity (48). Multiple sclerosis (MS), the most common idiopathic inflammatory neurodegenerative disease of the central nervous system, was also considered and can be associated with longitudinally extensive lesions such as the one in this case in up to 14% of children with MS (3, 49). However, our patient's single clinical event with one MRI lesion demonstrates neither dissemination in space nor time and does not meet IPMSSG criteria for pediatric multiple sclerosis (50). Furthermore, the chance that our patient experienced a clinically isolated syndrome preceding a future diagnosis of MS is <4% given negative CSF testing for oligoclonal bands (51). Systemic rheumatologic causes, such as systemic lupus erythematous (SLE), were ruled out because diagnostic clinical features were lacking and laboratory testing was negative. Additionally, lack of contrast enhancement of the LECL on MRI is inconsistent with the majority of reports of sarcoid myelitis or a covert spondylitic myelopathy (13, 14).

Acute disseminated encephalomyelitis (ADEM) is a poorly defined syndrome of multifocal CNS inflammation that represents 22–32% of acquired monophasic demyelinating syndromes in children and has a slight male predominance (52–54). It is a diagnosis of exclusion, and while typically seen as a post-viral or post-vaccinal syndrome, it can occur in up to 25% of children with no predisposing history (55). Unlike in most inflammatory myelopathies, up to 61.5% of ADEM patients have normal CSF findings, consistent with our patient's workup (56). A handful of cases of ADEM isolated to the brainstem and spinal cord, including one with MRI findings of LECL restricted to the anterior horns, have been reported (23, 25, 57). Furthermore, ADEM is known to more often involve the gray matter than either MS or NMOSD (58). While some key differentiating features of ADEM such as encephalopathy, multifocal large brain lesions of the same age, and anti-MOG seropositivity were not seen in our patient, a diagnosis of spinal-cord isolated ADEM could not be effectively ruled out (56). Furthermore, approximately only 40% of ADEM cases demonstrate anti-MOG seropositivity (and only extremely rare cases have shown AQP-4 seropositivity) (59–61).

In a recent informatics-based study, Barreras et al. describe a predictive model for determining etiology of myelopathy based on the time differential from a patient's symptom onset to symptom nadir; in our patient's case, his symptom nadir fell between 2 and 3 days from symptom onset, placing him at Barreras' cutoff between acute and subacute presentations—supporting our differential diagnosis and suggesting either an ischemic (acute) or inflammatory (subacute) etiology for our patients LECL (62). Since AQP-4 and MOG-associated NMOSD, MS, and systemic autoimmune causes such as SLE or sarcoidosis were considered unlikely based on imaging and laboratory testing, clinical management of our patient was directed by existing evidence for treatment of acute disseminated encephalomyelitis (ADEM) and other acute inflammatory myelopathies more generally (while keeping in mind the possibility of a transient or spontaneously resolving ischemic etiology and maintaining the patient on aspirin throughout the admission). High-dose corticosteroids are widely considered first-line therapy for acute inflammatory central nervous system processes including ADEM and other autoimmune myelopathies (63–65). Furthermore, a large observational study comparing functional outcomes of children with ADEM strongly favored methylprednisolone over dexamethasone as the primary agent (66). In our patient, the urgency of a rapidly progressive quadriparesis with a likely immune-mediated etiology demanded adjuvant treatment. IVIG, well-described in autoantibody-mediated acute inflammatory demyelinating polyneuropathies, is frequently a first choice for adjuvant or second-line immunomodulatory therapy in inflammatory CNS disease (especially in pediatrics) because of its ease of administration through a peripheral intravenous line and its high tolerability (67, 68). However, more recent data suggests that IVIG is inferior to similar treatments in acute inflammatory myelopathy, such as plasmapheresis or cyclophosphamide (69, 70).

Additionally, plasmapheresis is acknowledged in the inflammatory myelopathy literature mostly for its use as a salvage therapy (71–73). There is increasing evidence, however, for a synergistic effect of plasmapheresis and corticosteroids when administered early in the disease course (70, 74). While the modulatory effect is temporary, plasmapheresis effectively eliminates pathogenic autoantibodies, inflammatory cytokines, and complement proteins, thereby enhancing the longer-lasting molecular and genetic effects of corticosteroids on macrophage activation and cytokine production. Targeting both innate and humoral immune responses with early administration of combination therapy proved successful in achieving a rapid functional improvement in our patient.

Multiple large cohort studies in NMOSD patients additionally support early intervention with apheresis therapies during flares and found that reduced delay to apheresis improved the chance of a complete therapeutic response (75, 76). Our experience and literature review suggest consideration of early initiation of plasmapheresis in inflammatory LECL, despite its logistical challenges. Further investigation of the merit of early combination therapy vs. corticosteroids alone as first-line treatment is warranted in this context.

## Conclusion

Swift and accurate diagnosis of the etiology of a T2-hyperintense gray matter LECL in pediatrics is a complex and critical task. Systematic literature review supports our experience that while inflammatory, infectious, and vascular etiologies should all be considered and worked up, aggressive immunomodulatory treatment should be maximized early in the disease course, especially in cases that present a time-consuming diagnostic challenge. Furthermore, plasmapheresis may contribute to more comprehensive functional improvement and should be further explored as part of a primary combination treatment strategy with high-dose steroids.

This case of LECL restricted to the anterior horns in a child emphasizes the use of an evidence based, thoughtful and multidisciplinary clinical reasoning team approach to inform the diagnosis, work up, and treatment of a rare, critical and elusive diagnosis in pediatrics where time is of the essence.

## Ethics Statement

Written informed consent regarding the submission and potential publication of this manuscript was obtained from the case study patient and his legal guardian. Additionally, consent for treatment was likewise obtained in the usual fashion during the course of the patient's hospitalization.

## Author Contributions

DG, FW, NI, TW, HJ, and SS drafted the manuscript. DG and FW designed figure and table. DG, FW, NI, TW, HJ, SS, DR, and GG edited and revised manuscript. DG, FW, TW, DR, and GG reviewed systematic literature. DG, FW, DR, and GG research team management and oversight.

### Conflict of Interest Statement

The authors declare that the research was conducted in the absence of any commercial or financial relationships that could be construed as a potential conflict of interest.
