# Supplementary material for: A Longitudinally Extensive Spinal Cord Lesion Restricted to Gray Matter in an Adolescent Male
Source: Front Neurol. 2019 Mar 20;10:270. doi: 10.3389/fneur.2019.00270 (PMC6435483; doi:10.3389/fneur.2019.00270)
Supplement: Supplementary file 1 [file Table_1.DOCX]

**Appendix A:** Systematic Literature Review PubMed Search Strategy

1. Child [MeSH]
2. Child [tw]
3. Children [tw]
4. Boy [tw]
5. Boys [tw]
6. Girl [tw]
7. Girls [tw]
8. Pediatrics [MeSH]
9. Pediatrics [tw]
10. Pediatric [tw]
11. Pediatric [tw]
12. 1 or 2 or 3 or 4 or 5 or 6 or 7 or 8 or 9 or 10 or 11
13. Spine [MeSH]
14. Spine [tw]
15. Spinal [tw]
16. Spinal cord [tw]
17. Spinal cords [tw]
18. Myelitis [MeSH]
19. Myelitis [tw]
20. 13 or 14 or 15 or 16 or 17 or 18 or 19
21. Gray matter [MeSH]
22. Gray matter [tw]
23. Anterior horn cells [MeSH]
24. Anterior horn [tw]
25. Grey matter [tw]
26. Owl’s eye [tw]
27. Owl eye [tw]
28. Snake eye [tw]
29. Anterior cord [tw]
30. 21 or 22 or 23 or 24 or 25 or 26 or 27 or 28 or 29
31. 12 and 20 and 30
